# Development and validation of Chinese compensatory health beliefs scale

**DOI:** 10.3389/fpubh.2024.1271409

**Published:** 2024-04-23

**Authors:** Hua Yu Shi, Ya Ru Zhang

**Affiliations:** School of Economics and Management, Shanghai Institute of Technology, Shanghai, China

**Keywords:** compensatory health beliefs, scale validation, Chinese cultural context, factor analysis, cross-cultural adaptation

## Abstract

Compensatory Health Beliefs (CHBs), the notion that healthy behaviors can offset the negative effects of unhealthy actions, have been widely explored in Western contexts. Yet, their relevance within the Chinese cultural milieu remains underexplored. The primary objective of this research was to develop and validate a Chinese version of the CHBs scale (CHBs-C), addressing the gap in the literature regarding the applicability of CHBs within the Chinese cultural context. A multi-stage translation (from English to Chinese) was first completed, and exploratory factor analysis was conducted (*n* = 476), yielding the 14-item scale (CHBs-C scale). Confirmatory factor analysis was conducted to assess the validity, and the 2-week test–retest reliability, internal consistency and convergent validity of the scale were also assessed (*n* = 308). Predict validity was verified through testing the relationships between CHBs and health behaviors and habits (*n* = 274). Factor analysis showed a different factor structure in Chinese context, with only one factor identical to the original version. The fitness index of the new factor structure was good. However, while the scale exhibited acceptable internal consistency and high test–retest reliability, its convergent validity and predictive validity was found to be limited on a general level. Despite this, significant correlations at the subscale level were identified, highlighting nuanced interactions between CHBs and specific health behaviors within the Chinese population. This study not only establishes the CHBs-C scale as a valid and reliable instrument for assessing compensatory health beliefs in China but also lays the groundwork for further exploration of its applications and the potential cultural adaptability of CHBs.

## Introduction

Compensatory Health Beliefs (CHBs) are defined as the notion that the negative consequences of unhealthy behaviors—especially those offering immediate gratification—can be offset by engaging in healthy activities (1, 2). A classic example of this mindset is thinking, “I can have a piece of cake now because I plan to exercise later.” Research has shown that in the context of preventing chronic diseases like cardiovascular disease and obesity, which are linked to unhealthy habits, individuals harboring CHBs tend to engage in compensatory health behaviors (3, 4). However, the appeal of CHBs may also lead some individuals to abandon healthy routines in favor of indulgent, unhealthy behaviors (4–8).

Rabiau et al. have proposed a theoretical model to explain CHBs, which can not only explain why people hold CHBs, but also predict people’s behavioral decisions when there is a conflict between emotional states (such as desire) and motivations (i.e., health goals) (2). According to the CHBs model, individuals confronted with such conflicts might adopt one of three strategies: resisting the temptation, altering their perception of risk or expected outcomes, or activating CHBs. Engaging in CHBs allows individuals to succumb to immediate desires while planning to counteract the negative effects later, essentially rationalizing their unhealthy choices as temporarily acceptable. Although the original purpose of CHBs theory is to further improve the intention of health compensation behavior and promote the appearance of health behavior by guiding the public’s CHBs, the actual occurrence of compensation behavior is still full of great uncertainty.

In recent years, the ‘Healthy China 2030 Strategy’ has underscored the importance of health in China, a focus further intensified by the COVID-19 pandemic in 2020. Despite this, the constant presence of temptations in daily life has sparked a surge in CHBs, particularly among China’s post-90s generation, who are addicted to ‘punk health maintenance’. For them, ‘going to the gym only after overeating’, ‘having beer with wolfberry and cola with codonoposis’, etc. seem to become a fashion trend. Faced with this phenomenon, the strategy of promoting public health by arousing CHBs and encouraging compensatory behavior has been questioned.

Addressing these concerns, scholars have conducted extensive empirical research, including the development and validation of CHBs scales and their specific subscales (1, 9–12). Most of these studies have utilized the CHBs scale developed by Knäuper et al. (1). According to their factor analysis results, Knäuper et al. demonstrated that the CHB construct composed four factors, i.e., (1) substance use, including six items summarizing compensatory behaviors related to drinking, coffee and smoking; (2) sleep/eating habits, including four items reflecting beliefs related to behaviors that can make up for sleep loss, skipping breakfast, and eating freely at dinner; (3) stress, including four items related to behaviors to compensate for the negative effects of stress; (4) weight management, including three items related to behaviors that can compensate for high calorie intake. The Cronbach’s α value of the overall CHBs scale was 0.80, and the retest reliability was 0.75 (*n* = 141), indicating that the overall CHBs scale had high stability in a long period of time.

As interest in CHBs grows, researchers have tested the reliability and validity of CHBs subscales and their revised version across various health behaviors such as diet (3, 6, 13, 14), exercise (12, 15), tobacco and alcohol intake (16–18). The existing results showed that the reliability and validity of the CHBs scale and the subscales were good, which could be used to predict the possibility of the occurrence of individual unhealthy behaviors. However, the empirical tests of these scales have mainly focused on European and North American cultural contexts, and the Chinese population has not been discussed in depth. Our study aims to fill this gap by developing a Chinese version of the CHBs scale and assessing its psychometric properties. Unlike prior research that primarily focused on CHBs’ behavioral implications, our study contributes to the theoretical understanding by examining the cultural adaptability of CHBs, exploring how cultural nuances influence the manifestation and operationalization of CHBs, thereby enriching the theoretical discourse on health beliefs across diverse cultural landscapes. Firstly, with reference to the English version of CHBs scale, the Chinese version is determined, and factor analysis is carried out in the context of Chinese culture. Then, the reliability and validity of the overall scales and subscales are testified according to the online survey results. Finally, the structural differences between CHBs scale in Chinese cultural background and other cultural background are discussed. This exploration not only broadens the theoretical application of CHBs but also provides a foundational step for future research to explore the intricate dynamics between cultural context and health behavior theories.

## Materials and methods

### Development of Chinese CHBs scale (CHBs-C)

The creation of the CHBs-C was grounded in the original 40-item scale developed by Knäuper et al. (1). The cross-cultural adaptation of the CHBs scale in this study followed the guidelines for the adaptation of health-related items in other contexts and/or cultures proposed by Guillemin et al. (19) and Beaton et al. (20), in addition to the back-translation process. Initially, two PhD students translate independently (one researcher and one non-researcher in the field). Subsequently, seven Chinese scholars and two PhD students from different disciplines (psychology and behavioral decision making) combined the translations into the Chinese CHBs scale. The expert group discussed the draft of the scale, compared each item in the English and Chinese versions, checked whether the word semantics were equivalent, whether the expression or terminology was equivalent, whether the translation was applicable to the Chinese cultural context, and whether there were differences in the meaning between concepts. For example, experts discussed the word *diet*. In both English and Western cultures, the word is mostly used to refer to dieting, or keeping weight under control by consuming fewer calories. But in Chinese context and culture, this word refers not only to dieting, but also to people’s daily eating plans or habits. Translation items had been adopted by consensus of the experts. Finally, 10 PhD students in related majors were invited to evaluate the comprehensibility and correctness of each item in CHBs-C scale, and the 40-item CHBs-C scale was determined through communication and discussion with two experts.

### Respondents and procedures

Sample 1 consisted of 476 participants, who were recruited via an online survey service, Credamo.^1^ Participants were briefed on the survey’s topic before being asked to anonymously complete an electronic questionnaire in April 2021. We conducted an item analysis for scale structure and exploratory factor analysis. Items not adhering to a normal distribution were removed, and the remaining items underwent principal axis factor analysis (PFA), resulting in the initial version of CHBs-C. Subsequently, 308 of these participants (Sample 2) retook the same questionnaire two weeks later to facilitate a confirmatory factor analysis (CFA), thereby finalizing the CHBs-C version. We explored differences in factor structures between the original CHBs and the CHBs-C. Assessments of internal consistency, test–retest reliability, and both convergent and discriminant validity were conducted to ascertain the scale’s stability and reliability. These analytical procedures largely mirrored those used by Knäuper et al. (1) and Kaklamanou and Armitage (10). Furthermore, to further assess the predictive validity of the CHBs-C among the Chinese population, we recruited an additional 274 participants (Sample 3) from the Credamo platform in August 2021, and conducted a correlation analysis.

### Measures

#### Compensatory health beliefs scale

CHBs in sample 1 and sample 2 were measured using Knäuper et al.’s original 40-item scale (1). CHBs in sample 3 were measured using the final version of CHBs-C for Chinese adults, which consisted of 14 items (see Table 1 for specific questions). Participants responded to each item based on five-point Likert scales, with one point indicating complete disagreement and five points indicating complete agreement.

**Table 1:** CHBs-C: item wording and factor loadings (*n* = 476).

Factor and items	1	2	3
Factor 1: exercising/eating/sleeping habits			
1. Not eating vegetables can be made up for by eating fruits.	**0.482**	0.099	−0.147
2. Exercising during the spring and summer can compensate for not exercising during the winter.	**0.602**	0.156	−0.118
3. Not working out regularly is OK if the person is active in everyday life.	**0.624**	−0.020	−0.066
4. Eating sweets is OK because it reduces stress.	**0.439**	0.019	0.207
5. If one exercises, then one can eat without much restriction.	**0.602**	0.074	−0.006
6. Eating junk food is OK if the person exercises regularly.	**0.637**	0.071	−0.014
7. It is OK to go to bed late if one can sleep longer the next morning (only the number of hours count).	**0.700**	−0.154	0.091
8. Too little sleep during the week can be compensated for by sleeping in on weekends.	**0.444**	−0.003	0.176
Factor 2: drinking/smoking			
9. The bad effects of smoking and drinking can be reduced by getting a good night’s sleep.	0.010	**0.556**	0.110
10. Red wine counteracts the effects of fatty food.	0.066	**0.602**	0.028
11. Fresh air counteracts the bad effects of smoking.	0.042	**0.651**	−0.047
Factor 3: stress			
12. The bad effects of stress can be made up for by exercising.	−0.176	0.262	**0.450**
13. A stressful day can be compensated for by relaxing in front of the TV.	−0.002	−0.019	**0.701**
14. Stress during the week can be made up for by relaxing on the weekend.	0.141	−0.083	**0.543**

Loadings were taken from the pattern matrix. Loadings in bold are values above 0.40.

#### Goal conflict and health motivation

Goal conflict was assessed using Goal Conflict Scale (21), as well as measures concerning the importance of health goals and hedonic goals (1, 22). Measures related to risk perception, health motivation and hedonic motivation were adapted from existing studies (1, 11, 23). All items were assessed on five-point Likert scales.

#### Health behaviors and habits

Health behaviors and habits were assessed using measures adapted from Downey and Chang’s and Bishop and Yardley’s scales (24, 25). Eight items measured about sport and exercise, regular sleep schedule, regular diet, balanced nutrition, work and rest, getting rid of disease-causing habits, relieving stress, good mentality. All items were assessed on five-point Likert scales.

### Statistical analyses

Descriptive statistics were used to describe the sample characteristics and calculate the preference percentages. Firstly, frequency distribution, item discrimination test, inter-item and item-total correlations, and principal axis factor analysis (PAF) was performed, to reduce the number of items and identify the initial version of the CHBs-C. Kruskal–Wallis tests were applied for the comparison of independent mean values, based on whether there was a normal distribution or not. Secondly, a validity assessment was carried out through CFA to confirm the validity of the CHBs-C and finalize its version. Diagonally weighted least square (DWLS) was chosen, because it was confirmed to provide more accurate parameter estimates and more robust fit indices when dealing with non-normality and different data type (26–28). We assessed the model’s fit using a three-step process recommended by Kline (29) and Stone (30). The model fit indices utilized encompassed the scaled Chi-square (*χ*^2^), degree of freedom (df), scaled *p*-value, scaled Standardized Root Mean Square Residual (SRMR), number of correlated residuals with an absolute value above 0.10, and number of standardized residuals above 1.96 (29–33). Thirdly, internal consistency reliability was evaluated utilizing McDonald’s ω (34, 35), while Spearman correlation analysis was employed to assess the test–retest reliability. Finally, convergence validity was evaluated using the reliability combination (CR) value and average extraction variance (AVE) value, and the square root of AVE and correlation values were used to evaluate the discriminant validity. Predictive validity was examined through Spearman’s correlation analysis between the CHBs-C and constructs including goal conflict (21), the significance of health goals and hedonic goals (1, 22), risk perception, health motivation, hedonic motivation (1, 11, 23), as well as health behaviors and habits (24, 25).

All data analysis was performed using SPSS 22.0 and R version 4.3.1 software (36).

## Results

### Descriptive statistics

In sample 1, 44.1% of the participants were female (male, *n* = 266; female, *n* = 210). As shown in Table 2, most of the respondents were aged from 21 to 30 (46.8%, *n* = 223) and 31 to 40 (41.6%, *n* = 198), and the majority of the respondents’ monthly income level concentrated in 2001 to 10,000 yuan (68.5%, *n* = 326). In sample 2, a total of 57.8% were female (*n* = 178) and 42.2% were male (*n* = 130). Participants’ ages ranged from 21 to 30 (46.1%) and monthly income level between 6,001 and 10,000 yuan (39%) held the majority share. In sample 3, 45.6% (*n* = 125) of the participants were female, 55.5% (*n* = 152) were between the ages of 21 and 30, and 31.8% (*n* = 87) earned between 6,001 and 10,000 yuan a month.

**Table 2:** Demographic characteristics of participants.

Variable		Sample 1 *n* = 476 (%)	Sample 2 *n* = 308 (%)	Sample 3 *n* = 274(%)
Gender	Male	266 (55.9)	130 (42.2)	149 (54.4)
	Female	210 (44.1)	178 (57.8)	125 (45.6)
Age (years)	≤20	16 (3.4)	10 (3.2)	8 (2.9)
	21–30	223 (46.8)	142 (46.1)	152 (55.5)
	31–40	198 (41.6)	128 (41.6)	101 (36.9)
	41–50	30 (6.3)	22 (7.1)	10 (3.6)
	≥51	9 (1.9)	6 (1.9)	3 (1.1)
Monthly income (CNY)	≤2000	33 (6.9)	22 (7.1)	18 (6.6)
	2001–6,000	145 (30.5)	98 (31.8)	83 (30.3)
	6,001–10,000	181 (38.0)	120 (39.0)	87 (31.8)
	10,001–20,000	91 (19.1)	52 (16.9)	69 (25.2)
	≥20,001	26 (5.5)	16 (5.2)	17 (6.2)

*n* indicates the size of the sample.

### Factor analysis

#### Analysis of item distribution

Like Knäuper et al. did within the item elimination (1), we made a criterion according to the results of item distribution. If the item distribution was skewed or unbalanced, it would be deleted, thereby retaining items that showed sufficient variability and would elicit a quite large range of responses. This process left 30 items out of the 40 items for subsequent analyses, and 12 were overlapped with the Knäuper et al.’s (1) 17-item scale.

#### Principle axis factor analysis

Preliminary analysis showed that all items were correlated with each other, and the correlation coefficient was below 0.90. In addition, the Kaiser-Meyer-Olkin statistic was greater than 0.50 (0.923) and Barlett’s sphericity test was significant (*p* < 0.001), indicating that the data were applicable to factor analysis. Then, consistent with Knäuper et al. (1), the 30 items were subjected to a PAF. The latent variables may be slightly correlated, so we employed an oblique rotation (promax) rather than orthogonal rotation (37, 38), resulting in six factors being extracted explaining 50.6% of the total variance. We scrutinized items that exhibited low loadings (< 0.40) onto a certain factor and dropped the redundant or relatively unimportant ones. This procedure led to the elimination of five items.

The factor analysis was repeated with the remaining 25 items, and four factors emerged explaining 47.4% of the total variance. Eleven items were removed due to the low loadings (< 0.40) and the same steps of analysis were repeated. Three factors were extracted from this last analysis accounting for 50.6% of the total variance, with loadings of all items on the related factor higher than 0.40 (see in Table 1). We labeled factors 1 to 3 according to the item contents in each factor: exercising/eating/sleeping habits, drinking/smoking, and stress, respectively. Each factor represented different health aspects in which consumers had the beliefs that it could be compensated.

Initial CHBs-C (see in Table 1) contained 14 items with Cronbach’s alpha values (0.801) above the recommended threshold value of 0.70 (38, 39).

#### Confirmatory factor analysis

After the PAF, we subjected the 14-item scale to a CFA through R version 4.3.1, using the data from sample 2 (*n* = 308). DWLS was chosen to account for the 3-factor model. In the first step, we fitted the model to the data. As shown in Table 3, the model failed the exact fit test, so it was tentatively rejected. In the second step, we examined standardized and correlational residuals. Based on the modification indices, we correlated error variances between items with nearly identical wording-specifically, item 7 (“It is OK to go to bed late if one can sleep longer the next morning (only the number of hours count)”) and item 8 (“Too little sleep during the week can be compensated for by sleeping in on weekends”)-or that addressed the same concept, such as item 4 (“Eating sweets is OK because it reduces stress”) and item 14 (“Stress during the week can be made up for by relaxing on the weekend”). Finally, we fitted the modified 3-factor model to the data. Results showed that the modified model was acceptable, the *p*-value (0.182) was higher than 0.05 (*χ*^2^ = 61.063, df = 52), and SRMR (0.042) was less than 0.08 (29–33, 40). Additionally, we evaluated the higher-order model. In the first step, it failed the exact fit test and the fit indices were the same as those of the original 3-factor model (see in Table 3), so we testified the modified model. The results (see in Table 3) showed that the modified model was acceptable. Therefore, we consider the total scale to be acceptable, and we concluded that the final 3-factor CHBs-C, to some extent, represented three dimensions of compensatory health beliefs in Chinese culture context.

**Table 3:** Model fit index (*n* = 308).

Measure	Value			
	3-factor model	Modified 3-factor model	Higher-order model	Modified higher-order model
*χ* ^2^	119.856	61.063	119.856	68.721
df	62	52	62	53
*p*-value	0.000	0.182	0.000	0.072
SRMR	0.063	0.042	0.063	0.045
Number of Correlated Residuals >0.10	9	3	9	5
Number of Standardized Residuals >1.96	15	6	15	8

*n* indicates the size of the sample.

### Reliability

#### Internal consistency

We estimated McDonald’s ω separately for the overall CHBs-C scale and each subscales using the data collected in samples 1 and 2 (represented in Table 4). The overall McDonald’s ω value of sample 1 and sample 2 were 0.779 (*n* = 476) and 0.709 (*n* = 308), respectively. Though the ω value was lower than 0.80, it was still acceptable, indicating good internal consistency. However, ω values of subscales were around 0.70, indicating low internal consistency.

**Table 4:** Internal consistency: CHBs-C subscales.

CHBs-C		
Subscales	McDonald’s ω	
	*n* = 476	*n* = 308
Factor 1: exercising/eating/sleeping habits	0.774	0.688
Factor 2: drinking/smoking	0.668	0.639
Factor 3: stress	0.576	0.630

*n* indicates the size of the sample.

#### Test-retest correlations

We calculated test–retest reliability (2-week interval) on data from sample 1 and sample 2. The Pearson correlation coefficient was 0.89 (*p* < 0.001, *n* = 308), indicating high test–retest reliability.

### Convergent validity

As shown in Table 5, the average extraction variance (AVE) value was lower than 0.50 for all factors, and the reliability combination (CR) value for factor 1 and 2 was higher than 0.70, but the other one was lower than 0.70, which not confirmed the good convergent validity. However, the square root of AVE is higher than the correlation value with other factors, so the discriminant validity of the CHBs-C was confirmed.

**Table 5:** Convergent validity and discriminant validity of CHBs-C (*n* = 308).

Variable	CR	AVE	Factor 1	Factor 2	Factor 3
Factor 1	0.783	0.355	0.596		
Factor 2	0.706	0.448	0.204**	0.669	
Factor 3	0.679	0.414	0.086**	0.118**	0.643

**p < 0.01.

### Predict validity

#### Correlations with goal conflict and health motivation

Table 6 displays the inter-correlations among CHBs-C and goal conflict perception, health goals, hedonic goals, risk perception, health motivation and hedonic motivation. Results showed that ‘exercising/eating/sleeping habits’ significantly negatively correlated with health goals (*r* = −0.12, *p* = 0.047, *n* = 274), and health motivation (*r* = −0.17, *p* = 0.005, *n* = 274), though relatively weak. This means that the more exercising/eating/sleeping is believed to be compensatory, the lower degrees of health importance and motivation are perceived. As to the overall CHBs-C, there was none statistically significantly correlation.

**Table 6:** Correlations of CHBs-C scale with goal conflict and health motivation (*n* = 274).

	Exercising/eating/sleeping habits	Drinking/smoking	Stress	CHBs-C scale
Goal conflict perception	0.063	0.018	0.010	0.037
Health goals	−0.120^*^	0.012	0.051	−0.008
Hedonic goals	0.081	−0.033	0.071	0.043
Risk perception	−0.033	−0.029	−0.057	−0.057
Health motivation	−0.170^**^	0.112	0.092	0.057
Hedonic motivation	0.087	0.021	0.045	0.065

*p < 0.05; **p < 0.01.

#### Correlations with Chinese adults’ health behaviors and habits

Table 7 reports the inter-correlations among CHBs-C and Chinese adults’ health behaviors and habits. Consistent with other studies, some statistically significant correlations were found. Firstly, the overall CHBs-C scale correlated significantly with the good mentality (*r* = 0.123, *p* = 0.041, *n* = 274), showing that the more CHBs-C, the better the mentality. Besides, exercising/eating/sleeping habits significantly negatively correlated with the overall health intention (*r* = −0.176, *p* = 0.003, *n* = 274), and specific behaviors including regular sleep schedule (*r* = −0.128, *p* = 0.034, *n* = 274), regular diet (*r* = −0.240, *p* < 0.001, *n* = 274), and balanced nutrition (*r* = −0.150, *p* = 0.013, *n* = 274). This indicates that the more individuals believed that negative effects of lack of exercising, nutrients, or sleeping can be compensated, the lower the intention of health behaviors, the more irregular the sleep, the more irregular the diet, and the less balanced the nutrition. Thirdly, drinking/smoking and getting rid of disease-causing habits were significantly correlated (*r* = 0.134, *p* = 0.026, *n* = 274), meaning that believing that drinking/smoking could be compensated for was correlated with greater resolution to break the bad habits. Finally, stress was significantly associated with good mentality (*r* = 0.150, *p* = 0.013, *n* = 274), showing that greater compensation for stress was correlated with better mentality.

**Table 7:** Correlations of CHBs-C scale with health behaviors and habits (*n* = 274).

	Exercising/eating/sleeping habits	Drinking/smoking	Stress	CHBs-C scale
Sport and exercise	−0.039	0.075	0.018	0.042
Regular sleep schedule	−0.128^*^	0.026	0.042	−0.006
Regular diet	−0.240^**^	−0.042	−0.012	−0.112
Balanced nutrition	−0.150^*^	−0.010	−0.078	−0.096
Work and rest	−0.055	0.016	0.096	0.040
Getting rid of disease-causing habits	−0.102	0.134^*^	0.008	0.052
Relieving stress	−0.004	−0.108	0.096	−0.019
Good mentality	−0.010	0.084	0.150^*^	0.123^*^
Overall health intention	−0.176^**^	0.043	0.069	0.001

*p < 0.05; **p < 0.01.

## Discussion

This study evaluated the Chinese version of the CHBs scale in the Chinese cultural context through factor analysis, reliability analysis, convergent validity analysis and predict validity analysis. In this study, only three factors emerged, namely, exercising/eating/sleeping habits, drinking/smoking and stress, not providing much evidence of reliability or validity of the English 17-item CHBs scale.

The results of factor analysis showed that the CHBs-C scale had some similarity with the English version: the factor ‘stress’ were basically the same, and items related to sleeping habits in the English version all clustered in factor ‘exercising/eating/sleeping habits’. However, there were differences in the other dimensions. Specifically, items ‘It is OK to skip breakfast if one eats more during lunch or dinner’ and ‘Eating whatever one wants in the evening is OK if one did not eat during the entire day’ did not appear in CHBs-C scale due to low factor loadings (< 0.40). Instead, items ‘Not eating vegetables can be made up for by eating fruits’, ‘Eating sweets is OK because it reduces stress’, ‘If one exercises, then one can eat without much restriction’, and ‘Eating junk food is OK if the person exercises regularly’ loaded on factor ‘exercising/eating/sleeping habits’. This shift likely reflects the profound importance of food in Chinese culture, where meals are rarely skipped, and there’s a stronger inclination toward finding reasons to eat more and better.

Additionally, in our study, items ‘Not working out regularly is OK if the person is active in everyday life’ and ‘Exercising during the spring and summer can compensate for not exercising during the winter’ clustered in factor ‘exercising/eating/sleeping habits’. These results were partially consistent with conclusions of Kaklamanou and Armitage (10), though different with Knäuper et al. (1). One possible explanation is that at the beginning of 2021, the COVID-19 pandemic suddenly got worse, thus arousing the public’s attention to exercising (41). What is more, in such a special time, considering the inconvenience of certain sports and outdoor activities, various indoor activities had become popular on the Internet, which makes the Chinese believe that lack of exercising can be compensatory. Besides, most of our surveys were undertaken around April to August of 2021, in which time the epidemic situation had been improved in China. This may also awaken the belief that less exercises in winter can be compensated for doing more during the summer.

Third, the focus of factor ‘drinking/smoking’ in our study was the same with the factor ‘substance use’ in English version in essence, which means the Chinese also believed in that harmful effects of drinking alcohol and smoking cigarettes could be reduced through certain behaviors. Nevertheless, items in CHBs-C scale, focusing more on effortless behaviors, were totally different from those in English version. One possible interpretation may be that the popularization of the harmful effects of tobacco and alcohol in China is a little later than that in western countries, so Chinese residents are reluctant to expend too much energy responding to such risky behaviors. In the meanwhile, item concerned with coffee was not included in the CHBs-C scale. This result may be due to the fact that coffee is not consumed on a daily basis in Chinese culture and is considered more of a common drink than an addictive substance. Moreover, coffee has been a fashion trend among the Chinese young and middle aged, and they insist that the benefits of coffee far outweigh the disadvantages. Therefore, there is no need to compensate for drinking coffee.

To sum up, the differences in factor structure found in this study can be mostly attributed to the influence of cultural differences between China and foreign countries. Meanwhile, the similar factor structure also confirms that CHBs scale reflect the Chinese people’s beliefs about health compensation to a certain extent.

The study concludes that the CHBs-C scale demonstrates acceptable reliability and predictive validity for measuring CHBs in the Chinese context. Particularly, we have obtained a high Pearson correlation coefficient (*r* = 0.89) in the retest reliability analysis, indicating that the CHBs-C scale was very stable in assessing CHBs. The higher retest reliability of the CHBs-C scale in this study compared to the English version (*r* = 0.75) may be due to the shorter time interval between the two tests (only two weeks), while interval in English version was 4.5 to 5 months. Furthermore, though the English version of CHBs subscale is not fully applicable in specific areas, such as weight regulation, eating habits, and tobacco and alcohol intake, the predict validity of the new subscales are good. The findings suggest that higher CHBs related to exercising, eating, and sleeping correlate with fewer healthy behaviors, while those related to drinking, smoking, and stress correlate with more positive health attitudes. These results align with some existing research (1, 2, 9) but challenge the direct relationship between conflict and CHBs posited by CHB theory, indicating a need for further validation and potential theory optimization in specific contexts.

Our research marks a significant theoretical contribution to the field of health psychology by extending the application of the CHBs model to a non-Western context. Through the development and validation of the CHBs-C scale, this study illuminates the influence of cultural factors on health beliefs and behaviors. The findings suggest that the underlying structure of CHBs may vary across cultures, indicating a need for a more nuanced theoretical model that incorporates cultural variability. This work not only validates the CHBs model within the Chinese cultural context but also challenges and expands the existing theoretical framework by highlighting the role of cultural specificity. By doing so, it opens new avenues for theoretical exploration and practical application in the promotion of health behaviors across diverse populations. The adaptation and validation process undertaken in this study underscore the importance of cultural considerations in the theoretical development and empirical investigation of health beliefs, offering valuable insights for future research aimed at tailoring health interventions to specific cultural contexts.

### Limitations and future directions

Our findings have illuminated significant challenges in the internal consistency and convergent validity of the CHBs-C scale, as evidenced by lower omega (ω) values and AVE values. Several factors contribute to these challenges, offering invaluable insights for future research and scale development.

A key factor identified is the complex interaction between cultural nuances and the conceptualization of health beliefs within the Chinese context. The current items of the CHBs-C scale might not adequately reflect the intricate nature of health perceptions and behaviors influenced by cultural diversity. This gap poses significant challenges in ensuring the scale’s reliability and validity when adapting it across different cultural settings. Addressing this issue, future research should aim to expand the scale through qualitative research incorporating diverse focus groups. This would involve engaging with individuals from a wide spectrum of ages and income levels to identify underlying themes, which could then inform the development of new items. Such an approach is expected to not only broaden the scale’s dimensional coverage but also enhance its applicability and relevance to a more diverse segment of the Chinese population, thereby ensuring a more comprehensive and nuanced understanding of health beliefs within the cultural context of China.

Furthermore, the choice of a 0.4 cutoff for item deletion during the PAF process, inspired by the research of Knäuper et al. (1), might have contributed to the lower AVE values. While this criterion aimed to ensure the retention of significantly contributing items, it also highlights the delicate balance required in item selection to maintain construct integrity without overly narrowing the scale’s conceptual breadth.

Additionally, our sample’s demographic constraints, mainly encompassing individuals aged 21 to 40 with incomes ranging from 2001 to 10,000 yuan, highlight the importance of a more inclusive sampling strategy in future studies. Broadening the demographic representation is essential for enhancing the scale’s generalizability and validating its applicability across the diverse Chinese population.

In response to these findings, we advocate for a prudent approach to the scale’s application, especially recommending the initial use of the first factor as a provisional measure until further validation and refinement of the subsequent factors are achieved. This strategy underscores our dedication to the rigorous development of the CHBs-C scale. Future research should employ the CHBs-C scale in large-scale surveys to explore its factor structure and assess its reliability and validity among different demographic groups. Such endeavors are crucial for identifying the determinants of health beliefs and their correlations with unhealthy behaviors, offering a solid basis for targeted health promotion strategies in China. Through empirical studies, this tool can significantly advance our understanding of CHBs and their impact on behaviors such as Internet addiction (42) in the Chinese context, providing theoretical and practical insights for health promotion efforts.

## Data availability statement

The raw data supporting the conclusions of this article will be made available by the authors, without undue reservation.

## Ethics statement

The studies involving human participants were reviewed and approved by the Academic Board of the School of Economics and Management, Shanghai Institute of Technology. All participants provided their electronic informed consent to participate in this study, as the recruitment was conducted online.

## Author contributions

HYS: Formal analysis, Funding acquisition, Investigation, Methodology, Writing – original draft. YRZ: Funding acquisition, Project administration, Supervision, Writing – review & editing.

## Footnotes

^1^https://www.credamo.com
